# Effects of Songwriting Group Music Therapy Program among Informal Caregivers of Older Adults with Dependency

**DOI:** 10.3390/healthcare12171683

**Published:** 2024-08-23

**Authors:** Paula Pérez-Núñez, Oscar Martínez, Patricia Espinosa, Ane Perosanz, Irune García, Clare O’Callaghan

**Affiliations:** 1 Neuro-e-Motion Research Team, Faculty of Health Sciences, University of Deusto, Av. Universidades, 24, 48007 Bilbao, Spain; 2 Department of Medicine, St Vincent’s Hospital, University of Melbourne, 29 Regent Street, Fitzroy, VIC 3065, Australia; 3 Caritas Christi (Palliative Care Unit) and Psychosocial Cancer Care, St. Vincent’s Hospital, Fitzroy, VIC 3065, Australia

**Keywords:** informal caregivers, group music therapy, songwriting, HRQoL, psychological wellbeing

## Abstract

Informal caregivers of older people face demanding responsibilities that can negatively affect their wellbeing, highlighting the relevance of interventions that address their specific needs. This study aimed to assess the effects of a group music therapy program based on different songwriting approaches applied to informal caregivers of older adults with dependency. A sample of 113 caregivers participated, being assigned either to the intervention (*n* = 60) or the control group (*n* = 53). The intervention group caregivers received 10 weekly sessions of the songwriting program, while the control group continued with their usual care service. Psychological symptoms and health-related quality of life (HRQoL) were assessed at baseline and follow-up using the State-Trait Anxiety Inventory, Beck Depression Inventory-II, Zarit Burden Interview, and SF-36. Group × Time interactions revealed significant improvements in the intervention group compared to the control one in trait anxiety (*p* = 0.022), social functioning (*p* = 0.013), role limitations due to physical problems (*p* = 0.020), and physical component summary (*p* = 0.022). These findings provided valuable evidence on this novel intervention, showing positive effects for caregivers’ wellbeing. The current research emphasizes the importance of considering music therapy as a potential intervention among caregiver support programs.

## 1. Introduction

Due to global demographic trends of an ageing population and a rising demand for long-term care services, the role of informal caregivers is becoming critical, leading to increasing attention in healthcare research [1,2]. The definition of informal caregiver includes the person who provides assistance with activities of daily living to a relative with a chronic illness or disability without receiving any economical retribution [3]. Informal caregivers play a crucial role in providing care and support to older adults with various levels of dependency, enabling them to remain in their own homes and communities for as long as possible [4,5]. The demanding nature of caregiving responsibilities can significantly impact their wellbeing, particularly in the emotional dimension [6,7,8]. Understanding the challenges, needs, and experiences of informal caregivers is essential for developing effective interventions and support services tailored to their specific circumstances [9,10]. This includes addressing issues such as caregiver burden, stress, depression, and social isolation, which can impact not only on the caregiver’s health but also the care recipient’s [11,12,13].

In response to these implications, interventions focused on improving the wellbeing of caregivers have been explored, including the utilization of music therapy [14,15,16,17]. The concept of music therapy has been defined by the World Federation of Music Therapy [18] as “the professional use of music and its elements as an intervention in medical, educational, and everyday environments with individuals, groups, families, or communities who seek to optimize their quality of life and improve their physical, social, communicative, emotional, intellectual, and spiritual health and wellbeing. Research, practice, education, and clinical training in music therapy are based on professional standards according to cultural, social, and political contexts”.

Among the different music therapy studies focused on informal caregivers, the most common technique applied is group songwriting [17]. Songwriting in a therapeutic context refers to the creation of lyrics and/or music with the aim of addressing the psychological and health needs of an individual or group of people [19]. Between the strengths of songwriting, as a technique employed by clinicians, Baker [20] highlighted its versatility, its connection with culture and society, the combination between music and language, the fact that it involves therapeutic processes and invites a therapeutic relationship, and its nature as a medium for emotional expression.

The scientific literature has shown positive outcomes from the use of music therapy with informal caregivers. Through different methodology designs (i.e., qualitative, quantitative, and mixed methodologies), studies have reported decreases in anxiety and depression, enabling the expression of feelings and mood regulation, and a positive influence on wellbeing, stress, self-esteem, burden, and connection with others [21,22,23,24,25,26]. Despite these promising findings, the evidence is not conclusive enough since the number of studies that have applied controlled clinical trial designs is limited.

Music therapy research is increasingly emphasizing the relevant role of clinical trials, providing more objective evidence for the effectiveness of these interventions. Nevertheless, there is a lack of studies that specifically address the effects of music therapy interventions on informal caregivers. Baker et al. [27] presented a trial design as a feasibility study of implementing a group songwriting program with family caregivers of people living with dementia. This study reported that depression was a valid variable sensitive to change and had the potential to be considered in larger controlled trials. Ferguson [28] examined the effect of a music therapy songwriting intervention on the identification and utilization of coping strategies by caregivers of individuals with Alzheimer’s disease. The experimental group was able to identify and employ significantly more coping strategies between pre-test and post-test measures. Nevertheless, no significant differences were found between the data from the control and experimental groups.

There are some concerns identified in the literature review on this topic, which could lead to confusion, considering some studies as trials of music therapy applied to informal caregivers when they are not. On the one hand, most studies tend to focus on the care recipient, applying dyadic and joint interventions, with less attention given to informal caregivers, not focusing exclusively on family caregivers [29,30,31,32,33,34,35]. On the other hand, there are some clinical trials where it is not clear whether it is a music-based intervention or a music therapy intervention, finding an inconsistent and inaccurate use of terminology when referring to music therapy. This creates confusion and contributes to problems because “music therapy” refers to a profession, implying specific education, training, and credentials of the music therapist. Thus, we can find clinical trials about music applied to informal caregivers, but some of them could not be stated as music therapeutic interventions [34,35,36,37].

Considering the few studies found that evaluate the effects of a music therapy intervention on informal caregivers, it is considered relevant to contribute new clinical trials focused exclusively on caregivers. Thus, the purpose of the current study was to assess the effects of a structured songwriting group music therapy (SGMT) program in a sample of informal caregivers of older people with dependency. The main objective of the treatment was to improve their health-related quality of life (HRQoL), focusing on addressing emotional wellbeing through a group-based songwriting approach. The experience of the informal caregivers who participated in the intervention has been reported previously through a mixed methodology [38]. However, the effectiveness of the intervention could not be confirmed due to the absence of a control group. Therefore, in the present research, the hypothesis was that caregivers who completed the songwriting intervention would reduce their levels of anxiety, depression, burden, and improve their HRQoL compared to those in the control group.

## 2. Materials and Methods

### 2.1. Participants

**Figure 1:**
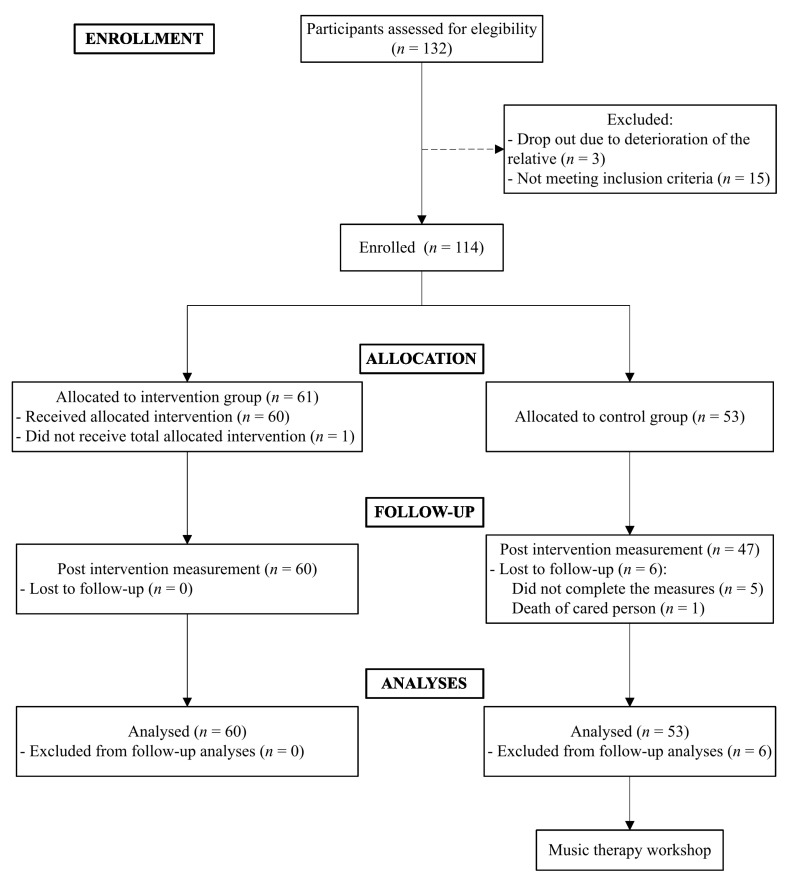
Participants flow diagram.

The sample of this study consisted of 113 informal caregivers of older adults aged between 41 and 87 years. Caregivers were recruited from different services and programs offered to support their needs from Biscay, Spain. A nonprobabilistic convenience sampling method was carried out. As Figure 1 illustrates, 18 of the 132 caregivers assessed for their suitability to participate in the study were excluded, most of them because they did not meet the inclusion criteria.

Participants were conveniently collected and allocated to the intervention group (*n* = 60) and to the control group (*n* = 53). Both groups’ sociodemographic data of the final sample are provided in Table 1. The inclusion criteria for both groups were as follows: (a) to be the main informal caregiver of an older person, (b) the care recipient was over 60 years of age, (c) caregiver had to be over 18 years of age, (d) attend a socio-sanitary center that provides support for informal caregivers, and (e) signed the informed consent form accepting the respective research condition. Participants were not included in the study if they met the following exclusion criteria: (a) not living in Spain, (b) below the age of 18, (c) presented uncompensated sensory and/or cognitive deficits that compromised the response to the assessment, (d) illiteracy, and (e) a diagnosis of any severe or serious mental illness.

### 2.2. Measures

Caregivers reported sociodemographic and caregiving data through a 35-item ad hoc questionnaire. Sociodemographic variables included age, gender, marital status, years of education, and occupation. In addition, data related to the caregiving context were collected (e.g., kinship, care hours, and dependency of the care recipient). The levels of functional dependency of the cared person were assessed through the Barthel index [39]. This test includes 10 items rated differently, using a 0-, 5-, 10-, or 15-point scale, ranging from complete dependence (unable to perform the task) to complete independence (able to perform the task without assistance). The global score ranges from 0 to 100, with higher scores indicating higher independence, providing a quantitative measure of the individual’s ability to carry out activities of daily living. The Spanish version of the questionnaire presented a 0.86–0.92 Cronbach’s alpha [40]. In this study, the Cronbach’s alpha coefficient was 0.91.

#### 2.2.1. Outcome Measures

##### Anxiety

The State-Trait Anxiety Inventory (STAI) [41] is a psychological questionnaire that assesses both state and trait anxiety levels of individuals. Therefore, this tool consists of two scales with 20 items each. The first subscale is the State Anxiety Scale (STAI-S), which evaluates current feelings of anxiety. The second one, the Trait Anxiety Scale (STAI-T), indicates individuals’ tendency towards anxiety across situations. All items are rated on a 4-point scale (from 0 to 3), and the total score ranges from 0 to 60, with higher scores indicating greater anxiety. The Spanish version of the STAI reported good psychometric properties, with a Cronbach’s alpha reliability of 0.90 for STAI-T and of 0.94 for STAI-S [42]. In the current study, Cronbach’s alpha coefficient was 0.92 for STAI-T and 0.95 for STAI-S.

##### Depression

The Beck Depression Inventory-II (BDI-II) [43] is a self-report scale designed to detect and assess the severity of depression over the last two weeks. It consists of 21 items rated on a 4-point Likert-type scale ranging from 0 to 3. The total score ranges from 0 to 63, where higher scores indicate more severe depressive symptoms. The Spanish version´s psychometric properties presented a Cronbach’s alpha of 0.89 [44]. This study obtained a Cronbach’s alpha coefficient of 0.90.

##### Caregiver’s Overload

The Zarit Burden Interview (ZBI) [45] assesses the levels of subjective burden experienced by informal caregivers providing care to patients with several medical conditions. It is a 22-item instrument rated on a 5-point Likert scale that considers different aspects of caregiver burden, where upper scores indicate higher burden. The Spanish version showed good internal consistency with a Cronbach’s alpha of 0.90 [46], obtaining the same coefficient in the present study.

##### HRQoL

The Short Form 36 (SF-36) [47] measures individual’s perception of their overall health and wellbeing across various physical and mental health domains. It is employed in clinical practice and research settings. This test consists of 36 questions, scored on Likert-type scales with response options ranging from 2 to 6 points. It assesses the health status of the person through eight dimensions: physical functioning, role limitations due to physical problems (role-physical), bodily pain, general health, vitality, social functioning, role limitations due to emotional problems (role-emotional), and mental health. These scales are combined into two summary measures: physical component summary (PCS) and mental component summary (MCS). Higher scores on each of the variables indicate better HRQoL. In relation to the psychometric properties of the Spanish version of the SF-36, a study reported good internal consistency, with Cronbach’s alpha between 0.70 and 0.90 in all dimensions of the SF-36 [48]. The Cronbach’s alpha coefficient in this study was between 0.77 and 0.91.

### 2.3. Intervention

The 10-session SGMT intervention program was conducted by a qualified music therapist who is also a psychologist. Each session had a duration of one hour and it took place once a week with small fixed groups of 4 to 7 caregivers. Since the intervention was carried out in different centers, the setting had to be as similar as possible. The rooms where the intervention was conducted had to have low reverberation and good ventilation and lighting. In addition, the same setting of instruments was offered, mostly small percussion ones, so the room had to be big enough and be practically empty and without decoration.

The process was divided into three phases: phase 1—“Getting to know each other” (sessions 1–3); phase 2—“Sharing and Creating” (sessions 4–7); and phase 3—“Saying goodbye” (sessions 8–10). The main technique used was songwriting, conducting different approaches depending on each phase: phase 1, group song parody songwriting; phase 2, individual song parody and; phase 3, group original songwriting. The song parody refers to rewriting all or part of the lyrics of a pre-composed song. In turn, the original songwriting involves the creation of completely original lyrics and music [20].

The SGMT intervention was designed to be conducted in a group format and was applied through a semi-directive approach, going from more to less directive throughout the phases. This approach was possible thanks to the versatility of the songwriting technique, employing a more structured type of songwriting (song parody) to end up with a less structured one (original songwriting). In order to prepare for or close the songwriting activity, secondary techniques were used such as clinical improvisation or receptive techniques (e.g., relaxation, listening, and discussion of meaningful songs, and listening for image evocation).

The first phase was characterized by greater structure and directivity, facilitated by the group-based song parody songwriting approach employed. This was a very structured songwriting approach, since a pre-selected theme was used. The objective of this first composition was to provide participants with a base of security and support, and enable self-confidence in their creation process. The structure was also reflected in the music, since a traditional well-known song (e.g., “La Bamba”) was selected by the group to provide the melodic framework. In the second phase, the song parody approach was maintained; however, participants worked individually, with the objective of expressing their individual needs. The process in this phase was less structured than in the earlier one, as caregivers were familiar with the activity. Furthermore, each caregiver chose the parodied song and the choice of the theme was semi-structured (each participant decided to whom and what they wanted to write). Finally, phase 3 was conducted employing a different approach: the “group original songwriting”. This third phase of songwriting consisted of an original composition since both the lyrics and the music were created by caregivers. The theme and music selection were free, providing more freedom and less structure. The role of the music therapist consisted of facilitating the composition process, guiding them from a more distant method (i.e., offering them a known structure for lyrics or creating together the base for the melody).

### 2.4. Procedure

Prior to the recruitment process, the ethical concerns of the study were addressed, including the approval of the Ethics Committee of the University of Deusto (ETK-42/20-21) in 2021 and the registration of the trial at ClinicalTrials.gov under the ID NCT06028815. Additionally, the study was conducted in accordance with the ethical principles established by the Declaration of Helsinki. Completing and signing the informed consent form was mandatory in order to participate. This document contained information about the project, including the evaluation details (variables, duration, and number of evaluations), benefits and risks of participating, confidentiality of data, and the statement that participation was voluntary and they could withdraw at any moment.

This study employed a longitudinal quasi-experimental design. A two-groups nonrandomized controlled trial design was used with measures taken at two time points: pre-test (i.e., baseline) and a post-treatment follow-up. It was a single-blind study because participants did not know which research condition they were receiving. The intervention group received the 10 weekly one hour sessions of SGMT, with participants distributed into 11 subgroups of four to seven persons each. The control group followed a treatment as usual condition, where caregivers remained on the existing care regimen in their respective centers, without receiving any additional service between measurements. After the follow-up assessment, control group participants were invited to participate in a one-day music therapy workshop, in order to experience a music therapy session, living and experiencing the different techniques that they knew theoretically in the informative talk.

The first author contacted 11 different social and health centers that offered support to caregivers of older people with dependency in Biscay. In collaboration with the psychologists of each center, informative talks were organized for the informal caregivers to introduce them to the project. Due to the demanding nature of caregiving and limited availability to move around, allocation to conditions was by convenience, considering the caregiver’s reference center. This allocation to groups was assigned by the researcher, taking into account the number of caregivers and the space availability of each center. Thus, when possible, a 10-session intervention (intervention group condition) was offered between the two time point assessments. Otherwise, caregivers received the same informative talk and the two psychological evaluations, providing individual reports, and complemented with the music therapy workshop.

Those caregivers who agreed to participate signed informed consent and received the initial assessment from the first author via email. “Qualtrics” platform was used to create this self-administered survey, including sociodemographic, caregiving data questions, and instruments that assessed outcome measures. The contact details of the researcher were provided in the informed consent form in case any questions arose during the completion of the survey. The survey completion duration was approximately 1 h. The data collection phase was conducted in several periods from September 2021 to March 2024. The participants in both groups completed outcome measures at the enrolment phase (1 week before the intervention) and immediately after the end of treatment (3 months after the first measure).

### 2.5. Statistical Analyses

The SPSS software (Statistical Package for the Social Sciences) version 27 was used to perform statistical analyses. The adjustment to the normal distribution of the data was analyzed for each of the variables studied, using the Kolmogorov–Smirnov test. This analysis was conducted for each group (intervention and control) and for each time of assessment. Descriptive statistics were used to report the characteristics of the sample, corresponding these variables to a normal distribution. Continuous variables were described by mean and standard deviation, and categorical variables by frequency and percentage. To examine the differences between the intervention group and the control group in sociodemographic data at baseline, the independent samples t-test and the chi-square test (*χ*^2^) were used for quantitative and categorical variables, respectively.

Regarding the outcome variables, global scores were calculated. For the SF-36, the syntax provided by the authors of the instrument was applied. Most of the outcome variables did not have a normal distribution in any of the combinations for further analyses (i.e., comparation between groups or time points), with the coefficient (K–S) being significant (*p* > 0.05). In order to analyze the differences in outcome measures between groups at baseline, the Mann–Whitney U-test was used. The Wilcoxon test was performed for intragroup analysis to assess the differences in outcome variables between pre-test and post-test measures.

Finally, a doubly multivariate repeated measures analysis of variance (ANOVA), also known as two-way or mixed-design ANOVA, was conducted to examine the effects of the interaction of group factor (between subjects) and time factor (within subjects) on anxiety, depression, overload, and HRQoL. First, the multivariate test (Wilks Lamda) provided the overall effect. Second, Group × Time interaction individual effects from univariate mixed-model analysis determined the SGMT intervention’s efficacy on outcome measures individually. Due to the lack of an equivalent nonparametric analysis, values were transformed into z-scores. The significance level was set at *p* < 0.05. Partial eta squared (*η*^2^*_p_*) was obtained as an indicator of the effect size, where *η*^2^*_p_* = 0.01 is considered small, *η*^2^*_p_* = 0.06 medium, and *η*^2^*_p_* = 0.14 large.

## 3. Results

### 3.1. Sample Characteristics at Baseline

The flow diagram for the study is presented in Figure 1. A total of 113 caregivers participated in the study, with 60 corresponding to the intervention group and 53 to the control group. Six participants from the control condition did not complete the post-test measure of the study. The mean ages of the caregivers were 66.27 ± 9.83 and 63.29 ± 9.93 years in the intervention group and the control group, respectively. Participants were mostly females (90% intervention and 84.9% control group), married (66.7% intervention and 60.4% control group), and sons or daughters of the care recipient (45% intervention and 54.7% control group).

An analysis of sociodemographic variables compared between the intervention and control groups showed that there were no significant differences between groups at baseline (Table 1). It was also found that there were no significant differences in variables related to caregiving, such as the care hours or the level of dependency of the care recipient (*p* > 0.05).

Table 2 reports Mann–Whitney U-test values, which showed no significant differences between groups prior to treatment in any of the outcome measures (*p* > 0.05). However, the intervention group scored higher in anxiety (STAI-S and STAI-T), depression (BDI-II), and subjective burden (ZBI). Regarding HRQoL, in some dimensions, the control group presented better scores, with the summary scores being very similar between both groups.

### 3.2. Intra-Group and Inter-Group Analyses Results

As summarized in Table 3, when comparing pre-test scores with post-test ones, the intervention group showed a statistically significant decrease in trait anxiety (*Z* = −3.26, *p* < 0.001, *r* = 0.30) and depression (*Z* = −2.56, *p* = 0.010, *r* = 0.23). Regarding HRQoL outcome variables, statistically significant improvements were found after the SGMT intervention in social functioning (*Z* = −2.36, *p* = 0.018, *r* = 0.22) and mental health (*Z* = −2.07, *p* = 0.038, *r* = 0.19). The effect sizes of these differences ranged from small to medium. The intervention group presented an improvement in the rest of the outcome variables, although not significant. Results obtained for the control group intra-group analyses showed that from pre-test to post-test measures, there was a significant worsening in the physical component summary (PCS) (*Z* = −2.06, *p* = 0.039, *r* = 0.21), with a small effect size. For the rest of the outcome variables, no significant changes were observed.

The doubly multivariate mixed ANOVA indicated an overall significant effect of Group × Time interaction with a Wilks Lambda = 0.791, F (14, 92) = 2.053, *p* = 0.028, *η*^2^*_p_* = 0.209. Univariate mixed-model repeated-measures ANOVA Group × Time interactions detailed in Table 4 revealed significant differences between the intervention and control groups in trait anxiety (F (1,105) = 5.433, *p* = 0.022, *η*^2^*_p_* = 0.05). Regarding HRQoL, interactions revealed significant differences between groups in the following scores: role-physical (F (1,105) = 5.589, *p* = 0.020, *η*^2^*_p_* = 0.051), social functioning (F (1,105) = 6.424, *p* = 0.013, *η^2^_p_* = 0.058), and PCS score (F (1, 105) = 5.371, *p* = 0.022, *η*^2^*_p_* = 0.049). The effect sizes of these differences were small to medium.

**Figure 2:**
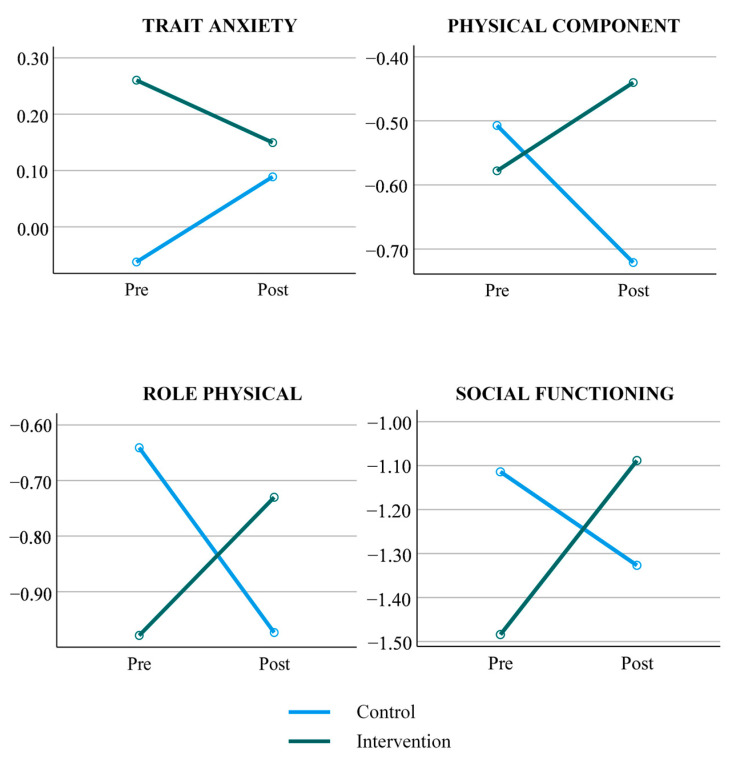
Significant outcome measures for Group × Time interactions. Green represents the intervention group and blue represents the control group.

As illustrated in Figure 2, there is a pattern of significant improvement in the mentioned outcome measures in the intervention group compared to the control group. Nevertheless, it should be highlighted that the control group declined over time in these outcome measures, presenting higher levels of trait anxiety, and lower levels of the HRQoL variables at the post-test.

## 4. Discussion

The aim of the current study was to evaluate the effects of a novel music therapy group-format intervention based on different songwriting approaches among informal caregivers of older adults. Specifically, it examined the effects of the SGMT intervention on outcome variables related to psychological wellbeing (i.e., anxiety, depression, and subjective burden) and HRQoL between the intervention and control groups. At baseline, the initial comparison of sociodemographic variables revealed no statistically significant differences between these groups, indicating the homogeneity of the sample. This lack of significant differences extended to caregiving-related variables, such as the number of care hours and the level of dependency of the cared-for individual. Further analyses supported this homogeneity on outcome measures.

The findings suggest that the intervention was effective in improving psychological outcomes and certain dimensions of HRQoL among caregivers. The significant reduction in trait anxiety and depression within the intervention group indicates the potential psychological benefits of the intervention. Furthermore, the improvements in social functioning and mental health scores highlight the intervention’s positive impact on HRQoL. These results, which were also reported in a previous work [38], are strengthened with the current study by the inclusion of the control group. While the intervention group showed the abovementioned improvements, the control group remained stable throughout the 3 months (the period between the two assessment points). The control group did not present significant differences in any of the outcome measures except for deterioration in the PCS. The control group’s decline in PCS and lack of improvement in other measures underscores the intervention’s relative efficacy. This decline also emphasizes the potential negative effects of continued caregiving without additional support or intervention, possibly exacerbating stress and physical health deterioration over time [6,12].

The interaction between groups, taking into account the different time points, revealed the effects of the intervention. The findings showed a positive impact on the trait anxiety variable and, regarding HRQoL, effects were found on physical role, social functioning, and PCS, with small to medium effect sizes. Regarding the remaining outcome variables, although they showed an improvement in the intervention group, the differences were not found to be statistically significant over time in comparison with the control group. Moreover, despite there being no significant differences between groups at baseline, it has to be considered that the higher scores of the intervention group at baseline in all the outcome measures could explain the lack of statistical significance shown in some of the inter-group analyses.

In accordance with the present results, it could be affirmed that this novel music therapy intervention based on different songwriting approaches addresses some of the previously reported psychosocial needs of informal caregivers, namely, trait anxiety, social functioning, and physical variables of HRQoL [6,7,8,11,12]. Although there are no similar controlled clinical trials to compare the results of the present study, other types of studies can be considered. The positive results of the SGMT intervention applied to informal caregivers are consistent with earlier research findings that showed positive changes in decreasing anxiety and fatigue, and improving HRQoL [22,34].

Concerning the effects sizes of the intervention found in this study, these are consistent with the previous literature in the topic of music therapy intervention effects. In other studies, moderate effect sizes have been found in anxiety, but small effect sizes have been found in relation to HRQoL [22,34], probably because of the complexity and multidimensionality of the construct. Furthermore, variables like anxiety and depression seem to be variables that are presented as more sensitive to change [22,27,34]. These small effect sizes that frequently appear on interventions with informal caregivers could be justified because of the great impact of caregiving on a caregiver’s life. This situation makes it difficult for interventions not maintained over time to produce high effects [49]. In the present study, one factor that could explain the small effect sizes and the nonsignificant differences in the other variables, like depression, is the variability of the sample, as they are informal caregivers of people with different pathologies. Nevertheless, these studies that found significant differences in depression used less conservative statistics [22,27].

The intervention also presented a positive effect on social functioning, which is a highly relevant aspect among the needs of informal caregivers [50]. However, this finding differs from other relevant studies, where, despite considering social aspects in the analyses, no significant differences were observed when comparing pre- and post-intervention measures. This does not necessarily mean that such changes did not occur, since when the literature review is extended to studies that employed qualitative methodologies, social-related variables are identified within the majority of the thematic analyses. For instance, Baker et al. [24] reported themes related to a sense of connection and reduction in loneliness. Similarly, García-Valverde et al.‘s [51] study identified a theme about their intervention that generated a sense of connection between group members, including three subthemes: a sense of belonging to a group, empathy and support, and the strength of the group. Furthermore, Klein and Silverman [24] also found group cohesiveness to be part of the thematic analysis of intervention results.

Considering all of this evidence, it can be concluded that qualitative studies collect the perceived experiences lived during the interventions, finding positive social outcomes. However, when analyzing the literature on intervention effects, this social aspect is not reflected in statistically significant quantitative results. This suggests that the SGMT intervention’s benefits extend beyond the intervention period, generalizing improvements in social functioning to a caregivers’ everyday life [52]. This highlights the importance of employing diverse research methods, as some positive outcomes might be missed or underreported, leading to the misconception of an intervention’s effectiveness when information has simply not been adequately collected and analyzed [53,54].

Despite the valuable insights gained from this study, several limitations need to be acknowledged. Firstly, there was a lack of randomization. Due to ethical considerations regarding participant burden and the need to ensure caregiver availability, a randomized controlled trial design was not feasible. Although this approach facilitated efficient participant recruitment and organization, it may have introduced selection bias and limited the generalizability of the findings. Nevertheless, blinding participants helped mitigate some potential biases. Another limitation of this study is the type of control group employed, where participants continued to receive the standard care from the services they usually attend. Ideally, a randomized controlled trial with an active control group, with participants receiving a different but structured intervention, would provide stronger evidence for the intervention’s effectiveness. However, the implementation of an active control group was not feasible due to resource constraints. Thirdly, the lack of follow-up data at 3 and 6 months limits the ability to determine if observed positive differences are sustained over time or new differences or effects sizes appears. This study was also limited by the dual role of the researcher, being also the music therapist who conducted the sessions. Another limitation to be pointed out is that despite this study providing results regarding the effects of the intervention, the reasons for this effectiveness were not studied. Finally, the limited availability of comparable studies in the literature makes it challenging to fully understand the significance of our findings.

To address these limitations, future research should explore alternative methodologies to study and understand the mechanisms of change behind these effects or secure additional resources to facilitate the inclusion of improvements in projects similar to the proposed one. Through this study, promising results were shown, and the significant improvements in the intervention group provide evidence for the effectiveness of this novel SGMT intervention, supporting the integration of such programs into regular caregiver support services. Therefore, further investment is necessary to include music therapy interventions and explore additional research possibilities, such as the inclusion of an active control group. Future research should also consider the long-term effects of the intervention and identify the specific components that are most beneficial. Additionally, studies could investigate whether similar interventions yield comparable benefits in more diverse caregiver populations or in different caregiving contexts.

## 5. Conclusions

In summary, this research constitutes a preliminary exploration that provides valuable information on the potential effects of the intervention. It represents a crucial step towards understanding the therapeutic potential of a structured SGMT intervention in supporting the overall wellbeing of informal caregivers of older individuals with dependency. This study highlights the relevance of targeting interventions to informal caregivers and addressing their own needs, implementing music therapy within the services directed to caregivers. The study’s results have significant implications for caregiver support programs, highlighting that interventions that focus on reducing anxiety and depression, and enhancing HRQoL, are crucial for supporting caregivers’ wellbeing. Furthermore, this SGMT intervention could be replicated and applied not only in the clinical field but also could continue its development through different research studies. Therefore, this study could make a substantial contribution to both the social and scientific international communities.

## Figures and Tables

**Table 1:** Baseline characteristics of the study sample.

	Intervention Group (*n* = 60)	Control Group (*n* = 53)		
	*M* (*SD*)	*M* (*SD*)	*t*	
	*n* (%)	*n* (%)	*χ* ^2^	*p*
Age (years)	66.27 (9.83)	63.23 (9.93)	−1.63	0.105
Sex			0.67	0.412
Women	54 (90%)	45 (84.9%)		
Men	6 (10%)	8 (15.1%)		
Marital Status			1.59	0.902
Married	40 (66.7%)	32 (60.4%)		
Living as a couple	5 (8.3%)	5 (9.4%)		
Divorced	3 (5%)	2 (3.8%)		
Separated	2 (3.3%)	1 (1.9%)		
Single	6 (10%)	9 (17%)		
Widow/er	4 (6.7%)	4 (7.5%)		
Years of education	13.97 (7.13)	14.02 (5.71)	0.04	0.965
Occupation			6.37	0.173
Employed	11 (18.3%)	19 (35.8%)		
Unemployed	7 (11.7%)	7 (13.2%)		
Retired	26 (43.3%)	19 (35.8%)		
Housework	13 (21.7%)	5 (9.4%)		
Other	3 (5%)	3 (5.7%)		
Kinship			1.53	0.676
Sons/daughters	27 (45%)	29 (54.7%)		
Spouses	26 (43.3%)	17 (32.1%)		
Brothers/sisters	4 (6.7%)	4 (7.5%)		
Other	3 (5%)	3 (5.7%)		
Care hours	42.07 (40.20)	51.55 (35.65)	1.32	0.190
Dependency—Barthel’s index	48.42 (30.22)	51.13 (29.98)	0.48	0.633

Note. *n* = number of participants; % = percentage of participants; *SD* = standard deviation; *t* = *t*-student; *χ^2^* = chi-square.

**Table 2:** Raw outcome measures scores for the intervention and control group at baseline.

	Intervention Group (*n* = 60)		Control Group (*n* = 53)			
	*M*	*SD*	*M*	*SD*	*Z*	*p*
Anxiety						
STAI-S	29.58	11.75	26.29	13.79	−1.52	0.128
STAI-T	28.68	10.37	24.63	12.68	−1.95	0.052
Depression—BDI-II	14.10	9.15	13.57	8.89	−0.57	0.571
Burden—ZBI	59.68	14.45	56.90	15.47	−0.81	0.417
HRQoL—SF-36						
Physical functioning	74.92	23.08	76.60	20.68	−0.17	0.867
Role-physical	48.75	42.79	59.91	45.02	−1.27	0.205
Bodily pain	50.92	25.38	51.30	24.21	−0.12	0.908
Social functioning	60.42	25.54	68.16	27.90	−1.59	0.111
Role-emotional	52.78	46.05	59.12	45.59	−0.68	0.498
Mental health	52.40	12.90	55.02	12.72	−1.08	0.279
Vitality	49.33	19.50	46.60	20.93	−0.99	0.318
General health	54.03	21.92	53.82	17.99	−0.06	0.956
PCS	44.22	10.61	44.91	9.89	−0.23	0.818
MCS	37.18	12.17	38.92	12.83	−0.78	0.437

Note. *n* = number of participants; *M* = mean; *SD* = standard deviation; STAI (S/T) = State-Trait Anxiety Inventory; BDI-II = Beck Depression Inventory II; ZBI = Zarit Burden Interview; SF-36 = Short Form 36; PCS = physical component summary; MCS = mental component summary.

**Table 3:** Intra-group analyses for outcome variables in the intervention and control groups.

	Intervention Group						Control Group	
	Pre-Test (*n* = 60)	Post-Test (*n* = 60)	*Z*	*p*	Pre-Test (*n* = 53)	Post-Test (*n* = 47)	*Z*	*p*
	*M* (*SD*)	*M* (*SD*)			*M* (*SD*)	*M (SD)*		
Anxiety—STAI								
STAI-S	29.58 (11.75)	27.57 (11.42)	−1.15	0.250	26.29 (13.79)	25.40 (13.66)	−0.79	0.430
STAI-T	28.68 (10.37)	25.95 (9.14)	−3.26	>0.001	24.63 (12.68)	25.04 (11.99)	−0.84	0.404
Depression—BDI-II	14.10 (9.15)	11.90 (8.39)	−2.56	0.010	13.57 (8.89)	13.72 (11.96)	−0.23	0.817
Burden—ZBI	59.68 (14.45)	58.13 (14.93)	−1.17	0.240	56.90 (15.47)	56.15 (17.07)	−1.06	0.288
HRQoL—SF-36								
Physical functioning	74.92 (23.08)	76.08 (21.94)	−0.34	0.737	76.60 (20.68)	75.04 (21.89)	−0.69	0.496
Role-physical	48.75 (42.79)	57.50 (41.25)	−1.34	0.179	59.91 (45.03)	48.94 (45.43)	−1.83	0.068
Bodily pain	50.92 (25.38)	55.13 (22.94)	−1.16	0.248	51.30 (24.21)	53.11 (25.98)	−0.46	0.647
Social functioning	60.42 (25.54)	68.33 (25.68)	−2.36	0.018	68.16 (27.90)	63.56 (29.93)	−1.15	0.250
Role-emotional	52.78 (46.05)	54.44 (45.91)	−0.29	0.765	59.12 (45.59)	66.66 (42.85)	−1.15	0.250
Mental health	52.40 (12.89)	55.47 (11.84)	−2.07	0.038	55.02 (12.72)	56.94 (12.01)	−0.65	0.519
Vitality	49.33 (19.49)	52.00 (18.55)	−1.42	0.155	46.60 (20.93)	49.57 (19.28)	−0.59	0.553
General health	54.03 (21.92)	54.93 (21.44)	−0.34	0.732	53.82 (17.99)	53.04 (17.42)	−0.18	0.855
PCS	44.22 (10.61)	45.59 (9.73)	−0.88	0.377	44.91 (9.89)	42.79 (10.54)	−2.06	0.039
MCS	37.18 (12.17)	38.76 (11.09)	−1.16	0.248	38.92 (12.83)	41.06 (11.49)	−0.53	0.597

Note. *n* = number of participants; *M* = mean; *SD* = standard deviation; STAI (S/T) = State-Trait Anxiety Inventory; BDI-II = Beck Depression Inventory II; ZBI = Zarit Burden Interview; SF-36 = Short Form 36; PCS = physical component summary; MCS = mental component summary.

**Table 4:** Mixed-model ANOVA results for outcome variables in the interaction of intervention and control group at baseline and follow-up.

		Intervention Group (*n* = 60)	Control Group (*n* = 47)	Time × Group		
		*M* (*SE*)	*M* (*SE*)	F	*p*	*η* ^2^ *_p_*
Anxiety—STAI						
STAI-S				0.166	0.683	0.002
	Pre	0.20 (0.12)	−0.02 (0.14)			
	Post	0.15 (0.13)	−0.02 (0.14)			
STAI-T				5.433	0.022	0.05
	Pre	0.26 (0.13)	−0.06 (0.14)			
	Post	0.15 (0.12)	0.09 (0.14)			
Depression—BDI-II				2.932	0.090	0.027
	Pre	0.04 (0.12)	0.01 (0.13)			
	Post	−0.04 (0.13)	0.15 (0.15)			
Burnout—ZBI				0.009	0.923	0.000
	Pre	0.18 (0.12)	0.05 (0.14)			
	Post	0.12 (0.13)	−0.00 (0.15)			
HRQoL—SF-36						
Physical functioning				1.132	0.290	0.011
	Pre	−0.41 (0.12)	−0.33 (0.14)			
	Post	−0.36 (0.12)	−0.40 (0.13)			
Role-physical				5.589	0.020	0.051
	Pre	−0.98 (0.16)	−0.64 (0.18)			
	Post	−0.73 (0.16)	−0.97 (0.18)			
Bodily pain				0.494	0.484	0.005
	Pre	−1.01 (0.12)	−0.97 (0.13)			
	Post	−0.86 (0.11)	−0.93 (0.13)			
Social functioning				6.424	0.013	0.058
	Pre	−1.48 (0.17)	−1.11 (0.19)			
	Post	−1.09 (0.18)	−1.33 (0.20)			
Role-emotional				0.415	0.521	0.004
	Pre	−1.19 (0.19)	−0.96 (0.22)			
	Post	−1.14 (0.19)	−0.73 (0.22)			
Mental health				0.829	0.365	0.008
	Pre	−1.04 (0.08)	−0.87 (0.09)			
	Post	−0.89 (0.08)	−0.81 (0.09)			
Vitality				0.231	0.632	0.002
	Pre	−0.79 (0.12)	−0.85 (0.13)			
	Post	−0.67 (0.11)	−0.78 (0.13)			
General health				0.219	0.641	0.002
	Pre	−0.64 (0.12)	−0.67 (0.14)			
	Post	−0.59 (0.12)	−0.68 (0.13)			
PCS				5.371	0.022	0.049
	Pre	−0.58 (0.13)	−0.51 (0.15)			
	Post	−0.44 (0.13)	−0.72 (0.15)			
MCS				0.010	0.922	0.000
	Pre	−1.28 (0.16)	−1.07 (0.18)			
	Post	−1.12 (0.15)	−0.89 (0.16)			

Note. *n* = number of participants; *M* = mean; *SD* = standard deviation; STAI (S/T) = State-Trait Anxiety Inventory; BDI-II = Beck Depression Inventory II; ZBI = Zarit Burden Interview; SF-36 = Short Form 36; PCS = physical component summary; MCS = mental component summary.

## Data Availability

The datasets generated and/or analyzed during the current study are not publicly available because they belong to the University of Deusto, but are available from the corresponding author (Paula Pérez Nuñez) on reasonable request.
